# Role of Ess1 in Growth, Morphogenetic Switching, and RNA Polymerase II Transcription in *Candida albicans*

**DOI:** 10.1371/journal.pone.0059094

**Published:** 2013-03-14

**Authors:** Dhanushki Samaranayake, David Atencio, Randall Morse, Joseph T. Wade, Vishnu Chaturvedi, Steven D. Hanes

**Affiliations:** 1 Department of Biomedical Sciences, School of Public Health, State University of New York, Albany, New York, United States of America; 2 Division of Genetics, Wadsworth Center, NY State Department of Health, Albany, New York, United States of America; 3 Mycology Laboratory, Wadsworth Center, NY State Department of Health, Albany, New York, United States of America; 4 Division of Infectious Disease, Wadsworth Center, NY State Department of Health, Albany, New York, United States of America; 5 Department of Biochemistry and Molecular Biology, State University of New York, Upstate Medical University, Syracuse, New York, United States of America; Stony Brook University, United States of America

## Abstract

*Candida albicans* is a fungal pathogen that causes potentially fatal infections among immune-compromised individuals. The emergence of drug resistant *C. albicans* strains makes it important to identify new antifungal drug targets. Among potential targets are enzymes known as peptidyl-prolyl *cis*/*trans* isomerases (PPIases) that catalyze isomerization of peptide bonds preceding proline. We are investigating a PPIase called Ess1, which is conserved in all major human pathogenic fungi. Previously, we reported that *C. albicans* Ess1 is essential for growth and morphogenetic switching. In the present study, we re-evaluated these findings using more rigorous genetic analyses, including the use of additional *CaESS1* mutant alleles, distinct marker genes, and the engineering of suitably-matched isogenic control strains. The results confirm that CaEss1 is essential for growth in *C. albicans*, but show that reduction of *CaESS1* gene dosage by half (δ/+) does not interfere with morphogenetic switching. However, further reduction of CaEss1 levels using a conditional allele does reduce morphogenetic switching. We also examine the role of the linker α-helix that distinguishes *C. albicans* Ess1 from the human Pin1 enzyme, and present results of a genome-wide transcriptome analysis. The latter analysis indicates that CaEss1 has a conserved role in regulation of RNA polymerase II function, and is required for efficient termination of small nucleolar RNAs and repression of cryptic transcription in *C. albicans*.

## Introduction

*Candida albicans* causes life-threatening fungal infections in hospitalized patients [1]–[3]. *C. albicans* is a commensal organism found on the human mucosal surface and is generally harmless in healthy individuals [4], [5]. However, *C. albicans* can cause systemic and sometimes fatal infections in immune-compromised individuals [6]–[8]. Life-saving therapies that require suppression of the immune system, *e.g.* organ transplantation and cancer chemotherapy, increase the risk for invasive candidiasis [9]–[12]. Premature infants, HIV-infected individuals, and individuals receiving prolonged intensive care treatment or antibiotic treatment are also vulnerable [13]-[16]. While potent antifungal drugs are available, the emergence of drug-resistant strains, especially against the widely used azole drugs is a growing problem [3], [17]–[21].

One strategy to overcome drug resistance while producing synergistic effects is the use of combination therapies that target distinct intracelluar pathways [22]–[27]. Toward this end, a number of different pathways are being investigated including those containing enzymes known as peptidyl prolyl *cis-trans* isomerases [28], [29]. PPIases catalyze the isomerization of the peptide bond preceding prolines within protein substrates [30], [31]. Three major families of PPIase have been described; cyclophilins, FK506-binding proteins (FKBPs) and parvulins (reviewed in [32]. All three families are conserved from yeast to humans. Inhibitors such as cyclosporin, which targets the cyclophilins, and FK506 or rapamycin, which target FKBPs, all show potent antifungal activity [33]–[36]. However, these drugs are also immunosuppressive via pathways that inhibit T-cell activation, an activity that could make individuals more vulnerable to fungal infections [37], [38].

An alternative PPIase target might be Ess1, a founding member of the parvulin class of PPIase, which is structurally distinct from the cyclophilins and FKBPs [39], [40] and whose human ortholog Pin1 is not known to be associated with the T-cell activation pathway. The first fungal Ess1 was discovered in *Saccharomyces cerevisiae* and shown to be essential [41]. Ess1 and Pin1 play critical roles in gene transcription by RNA polymerase II (pol II) [42]–[46]. Ess1 isomerizes peptide bonds within the carboxy-terminal domain (CTD) of Rpb1, the largest subunit of RNA pol II, and thereby controls binding and release of transcriptional co-factors [43], [47]–[49]. The X-ray structures of the *C. albicans* Ess1 protein and its human ortholog, Pin1 have been solved [40], [50], [51], and while the enzymes show overall similarity, there are key differences including a large solvent-exposed alpha-helix within a structured linker region that is present in the fungal enzyme but absent in the human enzyme. This helix has been hypothesized to play a role in fungal-specific functions, potentially by engaging in protein-protein interactions [50].

Homologs of Ess1 are found in all major pathogenic fungi that have been examined, including *C. albicans* [52], *Candida glabrata* (REFSEQ XP_445146), *Cryptococcus neoformans* [53], and *Aspergillus nidulans* (PinA) [54]. In *C. albicans* and *A. nidulans* Ess1 (PinA) is essential for growth [52], [54]. In *Cryptococcus neoformans*, Ess1 is not essential for growth, but is required for expression of virulence factors melanin and urease and for virulence in a mouse model [53].

In previous work, we isolated the *CaESS1* gene from *C. albicans* and showed it to be essential for growth in this organism using a novel temperature-sensitive mutant strategy [52]. Surprisingly, we found a heterozygous mutant strain (*Caess1*δ/*CaESS1*) to be defective for filamentation in various inducing media leading us to conclude that *CaESS1* gene dosage is important for morphogenetic switching [52]. However, several considerations led us to re-evaluate these findings. First, our *CaESS1* dosage experiments used the *URA*-blaster method [55], which has largely been superseded to avoid variations in virulence phenotypes due to expression of *URA3* from ectopic loci [56], [57]. Second, the importance of using reconstituted strains rather than parental strains as controls is now well-established [57],[58]. Finally, sequencing of the *C. albicans* genome [59] revealed a gene, *APE2* located very close to *CaESS1*, whose promoter might have been disrupted in our previous strain constructions.

We therefore re-examined our previous findings using newer information and more rigorous methods. The results showed that *C. albicans* Ess1 is essential for growth, but that in contrast to our previous report, *CaESS1* heterozygous mutants did not show a defect in morphogenetic switching, and instead the defect was traced to a host cell mutation(s). Potential effects of *URA3* marker gene placement were also ruled out. We also report results of a structure-function analysis of the CaEss1 linker α-helix, and describe a conditional-lethal readthrough allele of *CaESS1* that will be useful for further studies. Finally, results of a transcriptome analysis using high-throughput RNA-sequencing suggests that *C. albicans* Ess1, like its counterparts in budding yeast and humans plays a role in regulating RNA polymerase II function.

## Results and Discussion

### Reduction of *CaESS1* gene dosage does not affect filamentation phenotypes

**Figure 1:**
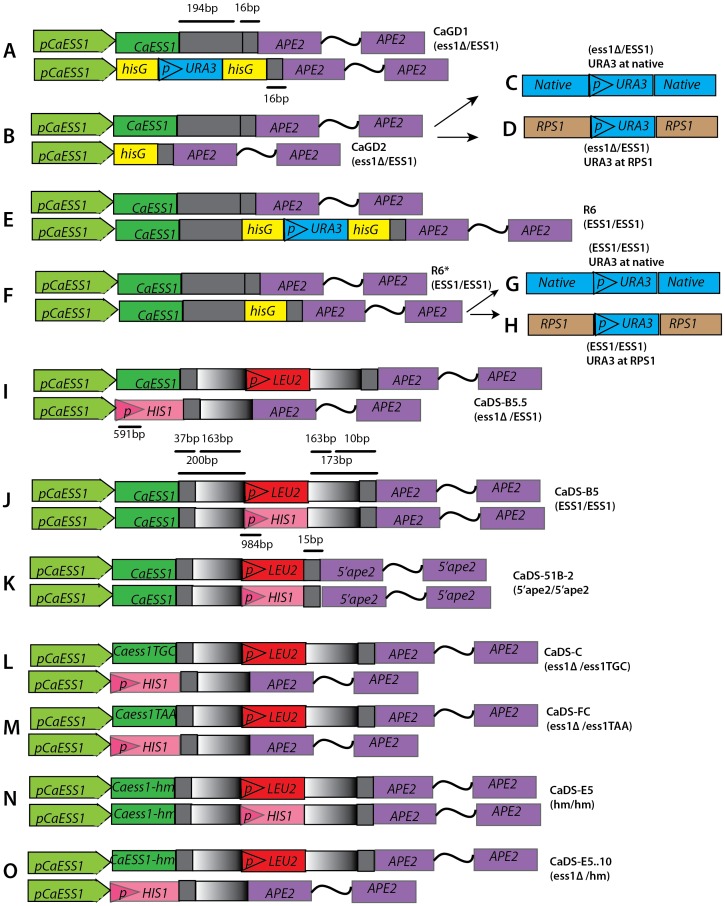
Schematic representation of the strains used in this study. Strains **A**–**H** are made in the CAI4(*) background using *URA3* as a selectable marker. Strains **I**–**O** are made in SN87 background with *HIS1* and *LEU2* selectable markers. The *CaESS1* coding region and promoter region (pCaEss1) are shown in green; the *APE2* gene is shown in purple. *APE2* gene has two exons and the wavy line represents the intronic region. *CaESS1* and *APE2* are 210 bp apart in the genome (grey). The *URA3* gene (blue) with the two flanking *hisG* direct repeats from *S. typhimurium* (yellow) is part of the construct used in the *URA*-Blaster method to target genes and later recycle *URA3*. *RPS1* is shown in brown, *LEU2* in red, *HIS1* in pink and (p) indicates promoter sequence. The sequence upstream of *APE2* that is repeated is shown in a gray-white gradient. The CAI4(*) indicates that this strain appears to have acquired a mutation that affects filamentation as per this study. Figure is not drawn to scale.

In previous work, we constructed a *C. albicans Caess1*δ/*CaESS1* heterozygous mutant strain in the CAI4 (ura^−^) parent strain using the *URA-*Blaster method [55] (**Figure 1A**). This strain, CaGD1, was defective for filamentation in Lee's, serum-containing and Spider media when compared to the “wild-type” control SC5314 [52]. The CAI4 (ura^−^) parent strain is a poorly-filamenting strain, presumably due to its uracil auxotrophy [60]. Instead, the parent strain of CAI4, clinical isolate SC5314, had been used as a control, because SC5314 is a uracil prototroph (ura^+^) and could therefore be compared to CaGD1 (ura^+^), even though SC5314 has two copies of *URA3* and CaGD1 has only one copy.

**Figure 2:**
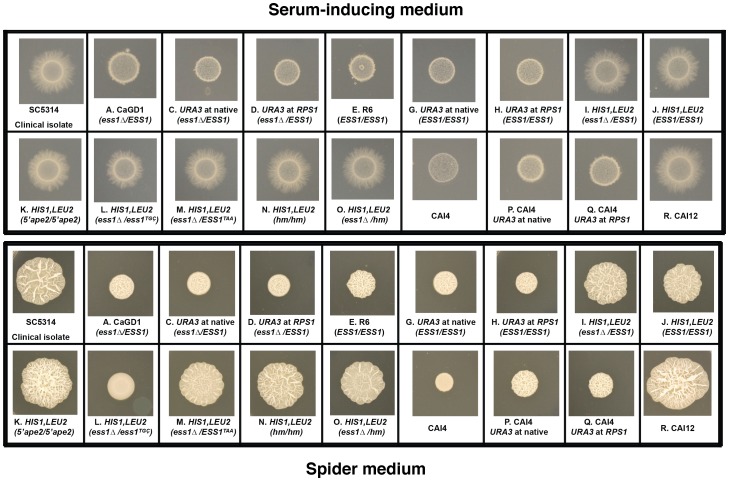
Filamentation of strains used in the study. Filamentation was tested on solid medium at 37°C. (**Upper panel**) Serum medium (4% FBS in 2% agar), (**Lower panel**) Spider Medium [80]. 2 µl of the 0.25 OD_600_ of fresh overnight cultures were spotted and grown for 4 days at 37°C before documentation. Small letters (A–O) within each panel refer to the constructs shown in Figure 1.

**Figure 3:**
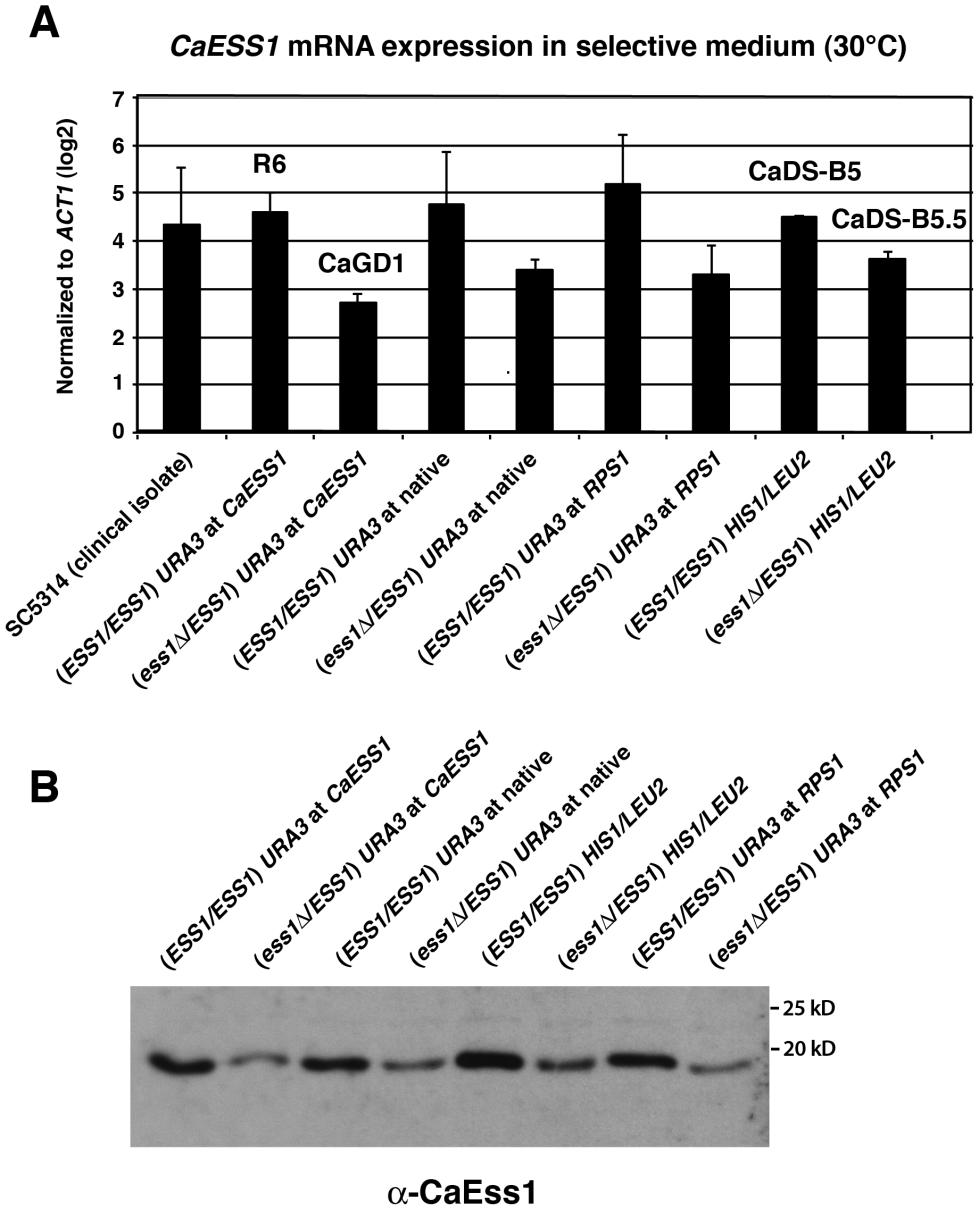
*CaESS1* is expressed at reduced levels in heterozygous mutants as expected. (**A**) Quantitative reverse transcription PCR (qRT-PCR) shows *CaESS1* mRNA expression levels in the indicated strains. (**B**) Western blot analysis showing the expression of CaEss1 protein in the indicated strains. A total of 3 µg of protein was used per lane. The blot was probed using anti-CaEss1 polyclonal antibody at a 1:500 dilution. CaEss1 is ∼19 kD. Strains correspond to those shown in Figures 1 and 2: (*ESS1*/*ESS1*) *URA3* at *CaESS1* is R6; (*ess1*Δ/*ESS1*) *URA3* at *CaESS1* is CaGD1; (*ESS1*/*ESS1*) *HIS1*/*LEU2* is CaDS-B5; (*ess1*Δ/*ESS1*) *HIS1*/*LEU2* is CaDS-B5.5.

Here, we generated a better control, isogenic to CAI4, but with one copy of the *URA3* gene placed at the *CaESS1* locus without disrupting it (**Figure 1E**). Surprisingly, this reconstituted control strain, R6 (ura^+^) (*CaESS1*/*CaESS1*), also did not filament on serum-containing medium or Spider medium even though both *CaESS1* alleles were left intact (**Figure 2E**). Filamentation on Lee's medium was also defective (data not shown). It is possible that the integrated *URA3* construct in R6 somehow lowered *CaESS1* expression levels in *cis*, but this was ruled out using quantitative reverse transcription real time PCR (qRT-PCR) and Western analysis, which shows the expected *CaESS1* RNA and protein levels in the heterozygotes (CaGD1) and wild-type control (R6) strains (**Figure 3**). Thus, placement of the *URA3* gene at the *CaESS1* locus or some other defect, but not *CaESS1* dosage, was likely responsible for the filamentation defect.

One possibility was that the *URA3* gene placed at the *CaESS1* locus interrupted the promoter region of the downstream gene, *APE2*. The *APE2* gene encodes an aminopeptidase that is thought to be secreted through the cell wall [61]. The design of our *URA3*-based *CaESS1* knockout and control constructs leaves only 15 bp of upstream sequence on one allele of *APE2*, leaving open the possibility that reduced expression of *APE2* was responsible for the filamentation defect in both strains (**Figure 1A, 1E**). As described in a later section, neither *URA3* nor *APE2* expression was involved.

To independently confirm that *CaESS1* gene dosage did not affect filamentation we took an approach that did not rely on *URA3* markers or disrupt the *APE2* promoter. We used a parent strain (SN87) in which *HIS1* and *LEU2* are used as selectable markers [62]. Extensive studies have shown that ectopic expression of these markers does not significantly affect the virulence functions [62]. Accordingly, we replaced one allele of *CaESS1* (with *HIS1*) generating a heterozygous strain (the other allele was marked with *LEU2*) (**Figure 1I**). We also constructed a control strain in the same background, but leaving both *CaESS1* alleles intact (**Figure 1J**). To avoid any expression defects in *CaESS1* or the downstream *APE2* gene, the constructs were designed to have a short direct-repeat sequence (163bp) flanking the markers such that the *ESS1* gene would have a total of 200 bp downstream of the coding sequence for transcription termination, and the *APE2* gene would have 173bp of promoter sequence left intact (**Figure 1I, J**; see also Materials and Methods). As before, qRT-PCR and Western analysis shows the expected reduction by about half of the expression of *CaESS1* mRNA and protein in heterozygous mutants (CaDS-B5.5) *vs.* the isogenic wild-type control (CaDS-B5 (**Figure 3**).

The *Caess1*δ/*CaESS1* heterozygous mutant strain and its *CaESS1*/*CaESS1* isogenic control showed no significant differences in filamentation phenotypes on inducing media (**Figure 2I, J**). In addition, no difference between these strains was observed in either germ-tube formation assays or in drug susceptibility growth assays using a number of commercially available antifungal drugs (data not shown). In summary, we find that the reduction of *CaESS1* gene dosage by half does not affect major virulence-related phenotypes of *C. albicans in-vitro*. We conclude that one copy of *CaESS1* is sufficient for growth and morphogenetic switching consistent with the finding that Ess1 in *S. cerevisiae* is present in excess under standard growth conditions [47].

### Neither *URA3* nor *APE2* levels are responsible for the filamentation defect in our CAI4 isolate

**Figure 4:**
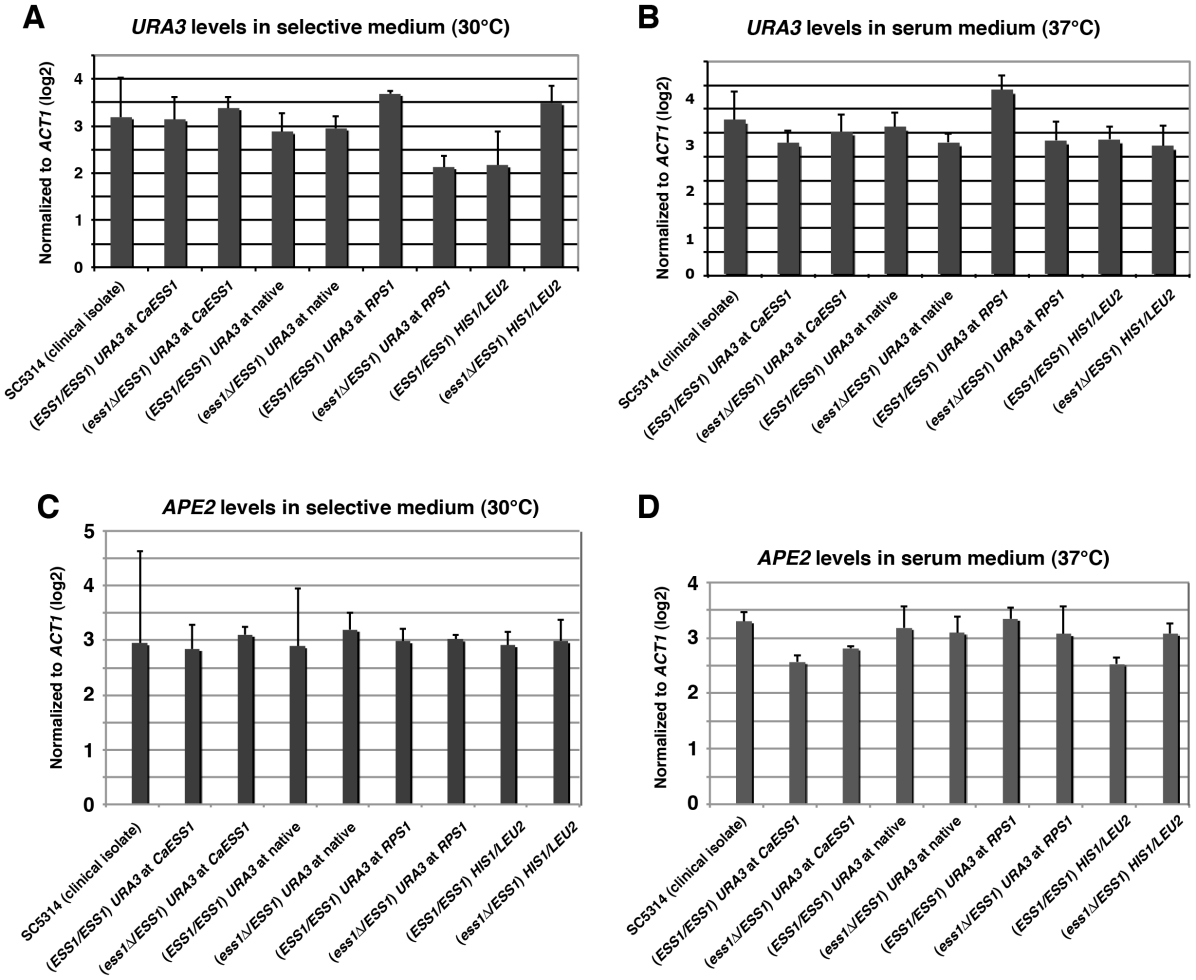
*URA3* and *APE2* mRNA expression levels are not significantly altered in heterozygous *ESS1* mutants and controls. qRT-PCR showing a quantitative measurement of *URA3* and *APE2* mRNA expression in the indicated strains. (**A**, **C**) Cells were grown at 30°C in complete synthetic medium (CSM), CSM minus uracil, or CSM minus histidine and leucine as appropriate for each strain. (**B**, **D**) Cells were grown at 37°C in serum-containing medium (10% FBS in YPD). Strains are as described in legend to Figure 3.

If *CaESS1* gene dosage was not responsible for the filamentation defects in our CAI4-derived strains, then what was responsible? To determine if ectopic *URA3* at the *ESS1* locus caused the filamentation defects in CaGD1 (*Caess1*δ/*CaESS1*) [52] and R6 (*CaESS1*/*CaESS1*) strains (**Figure 2A,E**), we created two additional sets of strains. In one set, the *URA3* gene in both the CaGD1 and R6 strains were removed and replaced into the native *URA3* locus (**Figure 1B,C & F,G**). In the other set, the *URA3* gene was removed and placed at the *RPS1* locus (**Figure 1B,D & F,H**). The *RPS1* locus is commonly used to express *URA3* and other genes to avoid positional affects [56], [57], [62]. We then used qRT-PCR to compare the levels of expression of *URA3* that resided at the *CaESS1*, native (*URA3*) or *RPS1* loci. This was done for cells grown in selective (uracil-deficient) media at 30°C and in 10% serum medium (10% FBS in YPD) at 37°C (**Figure 4A,B**). *URA3* expression was essentially the same regardless of where the gene resided, thus ruling out position effects on *URA3* expression as causing filamentation defects in our CAI4-derived strains. Moreover, placement of *URA3* at different locations had no effect on expression of *CaESS1* mRNA or protein (**Figure 3**).

Note, however, that all of the above strains carry one copy of the *APE2* gene with a truncated or *hisG*-disrupted promoter (**Figure 1A**–**F**). To test whether reduced *APE2* expression caused the filamentation defect, *APE2* expression was measured in multiple strains (corresponding to **Figure 1A**–**J**) using qRT-PCR. Expression was measured in cells grown in selective media (30°C) and in serum-inducing medium (10% FBS in YPD, 37°C), and no significant differences in *APE2* expression were detected (**Figure 4C,D**). These results suggest that the remaining copy of *APE2* with an intact promoter (strains in **Figure 1A**–**F**) compensated for the loss of upstream sequences in the second copy, or that the transcription is regulated from an internal promoter. In experiments done in the SN87 background using a strain with both *APE2* promoter regions truncated (*5′ape2/5′ape2*) (**Figure 1K**) neither the expression of *APE2* (data not shown) nor filamentation (**Figure 2K**) is affected. Results thus far indicate that *APE2* expression is not correlated with the filamentation defect in CAI4-derived strains.

Finally, to characterize our parental CAI4 isolate, which we now concluded was suspect, we placed *URA3* at the native locus or at the *RPS1* locus and tested its ability to filament. Analogous CAI4-derived strains (e.g. CAI12, **Figure 2R**) made in other laboratories using similar approaches [56], [63], [64] were able to filament, but the strains derived from our CAI4 isolate were not (**Figure 2P,Q**). Thus, not all CAI4 isolates behave similarly under inducing conditions, and we suspect that the isolate we used previously had acquired a background mutation(s) that resulted in a loss in the ability to undergo morphogenetic switching. These results explain the difference between our current results and those reported in Devasahayam *et al*., [52] with respect to *CaESS1* gene dosage and filamentation phenotypes. They may also explain the reduction in organ load in mice injected with SC5314 *vs*. CAI4-derivatives with mutations in *CaESS1* [50].

### *CaESS1* is essential for growth in *C. albicans*

Previously, a novel temperature-sensitive (*ts*) approach was used to demonstrate that *CaESS1* is essential for *C. albicans* viability [52]. In this approach one allele of *CaESS1* was deleted and the second allele was replaced with a form of *CaESS1* engineered to be conditional. Specifically, a substitution in a critical histidine residue in the *C. albicans* Ess1 active site (H171R) was generated based on a *ts*-mutation (*ess1^H164R^*) well characterized in *S. cerevisiae* [42]. Prior to its use in *C. albicans*, the *CaESS1^H171R^* allele was tested in complementation assays in *S. cerevisiae* and confirmed to be conditional; cells grew at 25°C and 30°C, but not at 37°C. When this allele was integrated into *C. albicans* CAI4, the resulting strain (*Caess1*δ/*Caess1^H171R^* strain) was *ts*-lethal: it grew at 30°C but not at 40°C, demonstrating that Ess1 is essential in *C. albicans*.

Here we sought to confirm, using this *ts*-strategy, that Ess1 is essential in another strain background, using a marker system other than *URA3*. Despite repeated attempts in the SN87 background, we were unable to generate a Ca*ess1*Δ/Ca*ess1^H171R^* strain, consistent with CaEss1 being essential and suggesting that in this strain background the *Caess1^H171R^* allele cannot support growth. However, this is a negative result, so we took another approach.

**Figure 5:**
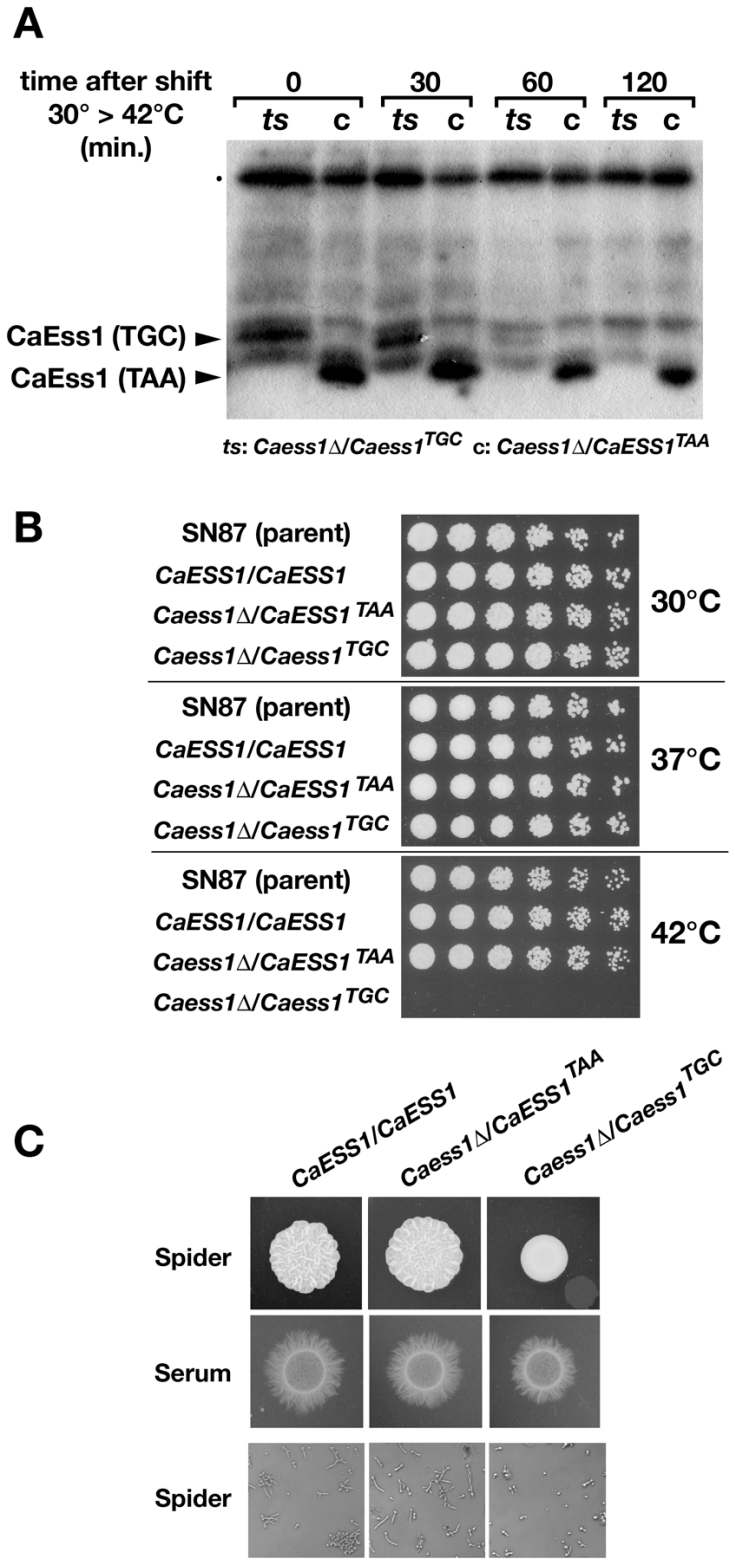
*CaESS1* is essential for the growth of *C. albicans*. (**A**) Western analysis of *C. albicans* whole cell extracts to detect expression of wild-type CaEss1 (TAA) and a larger protein product encoded by the *Caess1^TGC^* “readthrough” allele (TGC). The level of read-through protein is reduced significantly at 60 min and is nearly absent after 2 hrs at non-permissive temperature (42°C). A total of 7.5 µg of protein was used per lane. The blot was probed using anti-CaEss1 polyclonal antibody at a 1∶500 dilution. (**B**) Serial dilution (1∶3) of cells of the indicated genotype grown on solid medium (YPD) at different temperatures. The readthrough strain, *Caess1*Δ/*Caess1^TGC^* shows a clear temperature-sensitive phenotype at 42°C, but no growth defect at 37°C. (**C**) Filamentation on the indicated solid medium (4 days) (upper two rows), and germ tube formation in liquid Spider medium (2 hrs) (lower row). Upper two rows are reproduced from **Figure 2** for comparison. In (B) and (C), CaDS-B5 (*CaESS1*/*CaESS1*) is used as the wild-type, and is an isogenic control for both the TAA and TGC strains.

We noted that the analogous *S. cerevisiae ess1* ^H164R^ protein has<0.01% the catalytic activity of wild-type protein [47] and decided to generate an additional allele, again based on prior work in *S. cerevisiae* [42]. The allele we generated alters the termination codon (TAA to TGC), resulting in translational readthrough. In *S. cerevisiae*, analogous mutations resulted in longer, fusion proteins that rendered the corresponding strains *ts* (X. Wu and S. Hanes, unpublished). The *C. albicans* strain we generated here, CaDS-C (*ess1*δ*/Caess1^TGC^*; **Figure 1L**), which contains mutations in the stop codon (TAA to TGC) in CaEss1, encodes a protein that is larger than normal and is thermolabile at 42°C (**Figure 5A**). The level of mutant protein is only slightly reduced after two hours at 37°C (data not shown).

We compared the growth at different temperatures of the termination-mutant strain (CaDS-C) to an isogenic, reconstituted control strain in which the TGC readthrough codon was changed back to a TAA termination codon (CaDS-FC; *Caess1*δ*/CaESS1^TAA^*). Both strains grew at lower temperatures (30°C and 37°C), but only the wild type (reconstituted) strain grew at 42°C (**Figure 5B**). The results show that the *Caess1^ts(TGC)^* allele is unable to support sustained growth at the restrictive temperature demonstrating that CaEss1 is essential for growth in the SN87 strain background. Thus, using a distinct conditional allele (*Caess1^TGC^*) in a different strain (SN87) with different markers (*HIS1*, *LEU2*), we confirm that CaEss1 is essential in *C. albicans*.

### A large reduction in Ess1 levels reduces filamentation

In the course of characterizing the *ts*-lethal growth defect of the readthrough mutant strain (CaDS-C, *Caess1*δ*/Caess1^TGC^*), we noticed that at 37°C there is a reduction in filamentation relative to the control strain (CaDS-FC; *Caess1*δ*/CaESS1^TAA^*) under inducing conditions. This reduction is readily apparent for cells grown for 4 days on solid Spider medium (**Figure 5C** **, upper panels**). Even after 7 days, colonies were unwrinkled compared to controls (data not shown). To confirm this result, we examined cells grown in liquid Spider medium for 2 hrs. Again, we find that the readthrough mutant (*Caess1*δ*/Caess1^TGC^*) is reduced in germ-tube formation relative to reconstituted control strain (*Caess1*v*/CaESS1^TAA^*) (**Figure 5** **C.**). This is an intriguing result because we do not observe a growth-rate defect in the readthrough mutant when grown in standard (non-inducing) medium, even at 37°C. It seems likely therefore that the levels of Ess1 protein in this mutant strain are sufficient for standard growth but not for specialized functions such as the induction of filamentation pathways. This is consistent with findings in *S. cerevisiae* showing that Ess1 is present in vast excess in cells growing in standard media (YPD, CSM), but that under various stress conditions, a significant reduction in Ess1 levels rendered cells unable to grow [47].

### Structure-function analysis of the *C. albicans* Ess1

**Figure 6:**
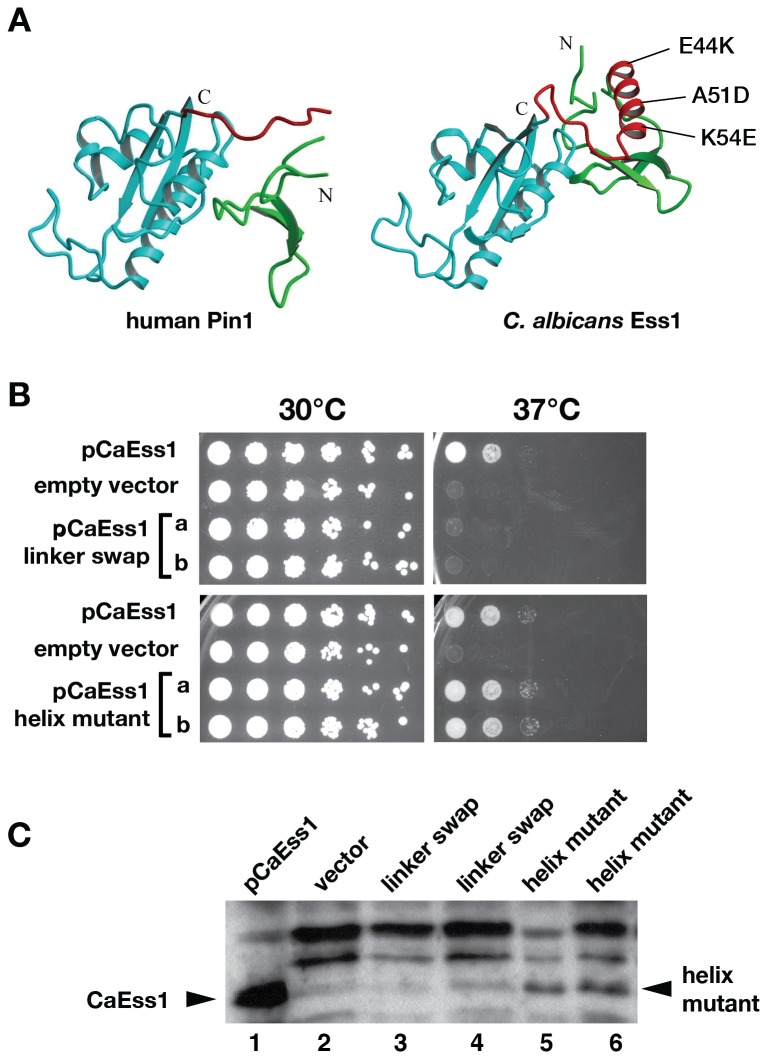
Structure-function analysis of CaEss1. (**A**) X-ray crystallographic structure of the human homolog, Pin1 [40] and CaEss1 [50]. Substitutions made in the structured linker α-helix of CaEss1 to construct the helix mutant (*hm*) strains are indicated. (**B**) Complementation of CaEss1 linker mutants in *S. cerevisiae*. Plasmids encoding the indicated mutant proteins (independent clone isolates of linker-swapped plasmid, pDS426(sw) and helix substitution plasmid, pDS426(pm)) were separately transformed into a *ts*-mutant strain of *S. cerevisiae* (*Scess1^H164R^*) [42]. Plasmids were constructed using a pRS426 backbone [88]. Serial dilution assays (1∶5) shows the growth of these independent transformants (a, b). Growth at 37°C, the restrictive temperature for the *S. cerevisiae ess1* mutant, indicates complementing activity. The CaEss1 helix substitution mutant complements but the linker swap mutant does not. pGDCaEss1 [52] (pRS426 backbone) was used as a positive control and pRS426 [88] was used as an empty vector control. (**C**) Western analysis of whole cell extracts of *S. cerevisiae* expressing the indicated CaEss1 proteins. A total of 15 µg of protein was used per lane and the blot was probed using anti-CaEss1 polyclonal antibody at a 1∶500 dilution. For the mutant strains, two independent clone isolates of pDS413(sw) (lanes 3 and 4) and pDS413(pm) (lanes 5 and 6) were transformed into *S. cerevisae* and analyzed. Plasmids were constructed using a pRS413 backbone [88]. The *S. cerevisiae* strain used (CBW22; [44] does not express endogenous Ess1 protein but is viable due to a suppressor mutation (*ess1*Δ*srb10*Δ). The linker-swapped protein appears to be absent, or present at a very low level compared to the vector control, while the helix mutant protein is easily detected. pGD-CaESS1 (pRS413 backbone) encoding the wild-type protein (Devasahayam and Hanes, unpublished) was used as a positive control, and pRS413 was used as the empty vector control.

The *C. albicans* Ess1 protein contains a highly-structured linker region that locks the WW and PPIase domains together [65]. Unlike the short, flexible linker in the human Pin1 protein, this linker also contains a prominent, solvent-exposed α-helix [50]. To help understand these key structural differences, we generated two mutations in the CaEss1 protein. First, we replaced residues 37–67 in *C. albicans* Ess1 protein with the flexible linker found in human Pin1 (residues 40–54), effectively generating a linker-swapped mutant. Second, we substituted three residues along the surface of the α-helix that would drastically alter the charge pattern it displays (E44K, A51D, and K54E; **Figure 6A**). As an initial test of protein function we tested their ability to complement a *S. cerevisiae ts* mutant (*ess1^H164R^*) at non-permissive temperature (**Figure 6B**) and to complement an *ess1*Δmutant using a plasmid-shuffle strategy (**Table 1**). The *C. albicans* linker-swapped mutant was unable to complement in either assay, whereas the helix mutant fully complemented in both assays.

**Table 1:** Function of *C. albicans* Ess1 linker mutants in *S. cerevisiae*.

Strain	# Patches	Ura(+)	Ura(−)	% Plasmid Loss	Interpretation
pRS413 (neg) control	400	400	0	0	No complementation
pCaESS1 (WT) (pos) control	216	144	72	33	Control level of complementation
pCaESS1 helix subsitution mutant	324	208	116	36	Full complementation
pCaESS1 linker-swapped	400	400	0	0	No complementation

Host strain (MATa ura3-1 leu2-3, 112 trp1-1 can1-100 ade2-1 his3-11, 15 [phi+] ess1Δ*TRP1*+YEpESS1) is an *ess1*Δ*TRP1* mutant of *S. cerevisiae* (Wu and Hanes, 2000, unpublished) covered by a 2 µM, *URA3* plasmid expressing *ESS1* (YEpEss1). Cells were transformed with the indicated plasmids (all 2 µM, *HIS3*) and plated on complete synthetic media (CSM) minus uracil (ura), histidine (his) and tryptophan (trp). Colonies were picked and passaged (20 ul into 3ml) for three successive overnights in liquid CSM minus his. After 3 days, individual colonies were patched onto CSM minus his trp plates, and replica-plated to CSM minus ura (for Ura+), 1mg/ml 5-FOA (for Ura−), and CSM minus his trp (for total patch number) and scored for uracil prototrophy after 1 day. For the helix subsitution mutants, three independent clone isolates of helix substitution plasmid, pDS413(pm) were tested. For the linker-swapped mutants, two independent clone isolates of plasmid, pDS413(sw) were tested.

To confirm that the mutant *C. albicans* Ess1 proteins were expressed and stable in *S. cerevisiae*, we performed Western blot analysis (using antibodies to *C. albicans* Ess1). The linker-swapped mutant was present at very low levels (**Figure 6C**), likely explaining its failure to complement in the functional assays. Due to its presumed instability, the linker-swapped mutant was not used for further studies. In contrast, the helix mutant was expressed at much higher levels, indicating the protein is stable consistent with its functionality (**Figure 6C**). That the helix mutant complements *S. cerevisiae ess1* mutants suggests it performs the essential catalytic function required for growth in this organism. This result is not entirely surprising given that the human and other vertebrate orthologs lacking this α-helix also rescue growth in *S. cerevisiae* [66]; Wilcox & Hanes, unpublished]. We interpret this result to mean either the α-helix is required solely for specialized functions, *e.g*. specific protein-protein interactions in *C. albicans* that might be lacking (or not revealed) in *S. cerevisiae*, or that it is not important for growth under standard conditions.

To further test the importance of this α-helix, we introduced the helix mutant (*hm*) alleles into *C. albicans* cells using the SN87 parent strain. Two strains CaDSE-5 (*hm/hm*) and CaDSE-5.10 (*Caess1*δ*/hm*) were constructed along with their respective isogenic controls. Both strains, CaDSE-5 and CaDSE-5.10 (**Figure 1N, O**) were tested for filamentation, germ tube formation and anti-fungal drug susceptibility profiles. No significant differences were seen in any of these assays (**Figure 2N, O** **, and data not shown**), suggesting the linker helix of CaEss1 is not important for morphogenetic switching or drug sensitivity in cultured cell assays. Thus, the prediction that the linker helix is important for fungal-specific functions has not yet been demonstrated. It is also possible that the triple mutation is not severe enough to inactivate its putative function(s), or that studies in animals models are needed to detect functional consequences of these mutations.

### A conserved role for CaEss1 in RNA polymerase II transcription

Orthologs of CaEss1 in budding yeast (Ess1) and humans (Pin1) regulate RNA pol II transcription [42], [45], [46]. In yeast, Ess1 coordinates the recruitment of co-factors required for proper RNA synthesis and processing [43], [49]. In this role, ScEss1 is required for efficient termination of at least two classes of RNA pol II products, small non-coding RNAs, *e.g.* small nucleolar RNAs (snoRNAs), and protein-coding mRNAs. ScEss1 is also important for repressing the transcription of cryptic unstable transcripts (CUTs) [49]. In *S. cerevisiae ess1* mutants, CUTs are detected throughout the genome, many of which are the same as those detected in RNA-decay mutants [43]. To determine whether CaEss1 plays an analogous role in *C. albicans*, we carried out a whole-genome transcript analysis in wild-type and CaEss1 mutant strains using high-throughput RNA sequencing (RNA-seq). The strains and conditions used are summarized in **Table 2**. A large number of mapped sequence reads were obtained (**Table 2**), indicating that our RNA libraries were of high quality.

**Table 2:** Summary of RNA sequencing experiment.

Strain	Harvest Conditions	# of Mapped Reads
*At Weill Cornell Medical College, NY*		
CaDS-B5 (*ESS1/ESS1*)	37°C serum induction (2 hr)	22,000,000
CaDS-C (*ess1*Δ*/ess1^TGC^*) *ts*-mutant	30°C>42°C temp.shift (1 hr) then serum induction (2 hr)	28,000,000
CaDS-FC (*ess1*Δ*/ESS1^TAA^*) control	30°C>42°C temp.shift (1 hr) then serum induction (2 hr)	21,000,000
*At Centrillion Biosciences, CA*		
CaDS-B5 (*ESS1/ESS1*)	37°C serum induction (2 hr)	22,000,000
CaDS-B5.5 (*ess1*Δ*/ESS1*)	37°C serum induction (2 hr)	21,000,000

Listed are the strains, growth conditions and number of total mapped reads per sample. For details, see Materials and Methods.

For each mutant and control pair analyzed, there was a significant change in the level of expression for between 2–4% of all transcribed genes (**Table 3**). This is only slightly less than that observed in budding yeast (wild-type *vs. ts*-mutant) using standard microarrays where 3–10% of genes were misregulated depending on the temperature [43]. The results indicate that in *C. albicans*, as in *S. cerevisiae* the expression of some but not all genes is adversely affected by mutations in Ess1. Raw sequence reads will be deposited in the European Nucleotide Archive (http://www.ebi.ac.uk/ena/). Analyzed datasets will also be submitted to the NIH Gene Expression Omnibus (GEO) (http://www.ncbi.nlm.nih.gov/geo/). In this report, we will focus on the comparison between the transcriptomes of the conditional (*ts*) readthrough mutant (*ess1*Δ/*ess1^TGC^*) and its isogenic control (*ess1*Δ/*ESS1^TAA^*), relative to the wild-type strain (*ESS1*/*ESS1*).

**Table 3:** Changes in gene expression based on RNA-sequencing results.

Test Strain (& growth conditions)	Relative to	Total affected genes (%)	Log_2_ fold-change	P value
**CaDS-C (** ***ess1*** **Δ** ***/ess1^TGC^*** **)** 30°C>42°C temp.shift (1 hr), serum induction (2 hr)	**CaDS-FC (** ***ess1*** **Δ** ***/ESS1^TAA^*** **)** 30°C>42°C temp.shift (1 hr), serum induction (2 hr)	2.24	6.4–0.99	0–0.002
**CaDS-C (** ***ess1*** **Δ** ***/ess1^TGC^*** **)** 30°C>42°C temp.shift (1 hr), serum induction (2 hr)	**CaDS-B5 (** ***ESS1/ESS1*** **)** 37°C serum induction (2 hr)	3.43	7.5–1.3	0–0.003
**CaDS-B5.5 (** ***ess1*** **Δ** ***/ESS1*** **)** 37°C serum induction (2 hr)	**CaDS-B5 (** ***ESS1/ESS1*** **)** 37°C serum induction (2 hr)	3.3	3.4–0.41	0–0.0017

The log_2_ fold changes are based on results from the Cuffdiff program in the Galaxy server. Cuffdiff was used to select significant gene expression changes depending on whether the *p* value was greater than the allowed false discovery rate after Benjamini-Hochberg correction for multiple-testing. The highest and lowest log_2_ fold changes of the genes considered to be significantly differentially expressed in each group are documented it in the table.

To determine whether CaEss1 functions in snoRNA termination, we obtained RNA-seq data from *ts*-mutant and control strains and examined the abundance of sequence reads at non polycistronc, independently-transcribed snoRNA genes of the box H/ACA-class [67]–[70]. In total, we examined expression of 26 H/ACA-class genes using Integrated Genome Viewer (IGV), and found that about one in five show evidence of readthrough as revealed by RNA-seq data. These genes included *SNR3a*, *SNR8a*, *SNR32a*, *SNR43a*, and *SNR189*) [67]. Primary transcripts of the other major class of snoRNAs (box C/D) are often encoded within other genes in *C. albicans* and were not examined here [68]–[70].

**Figure 7:**
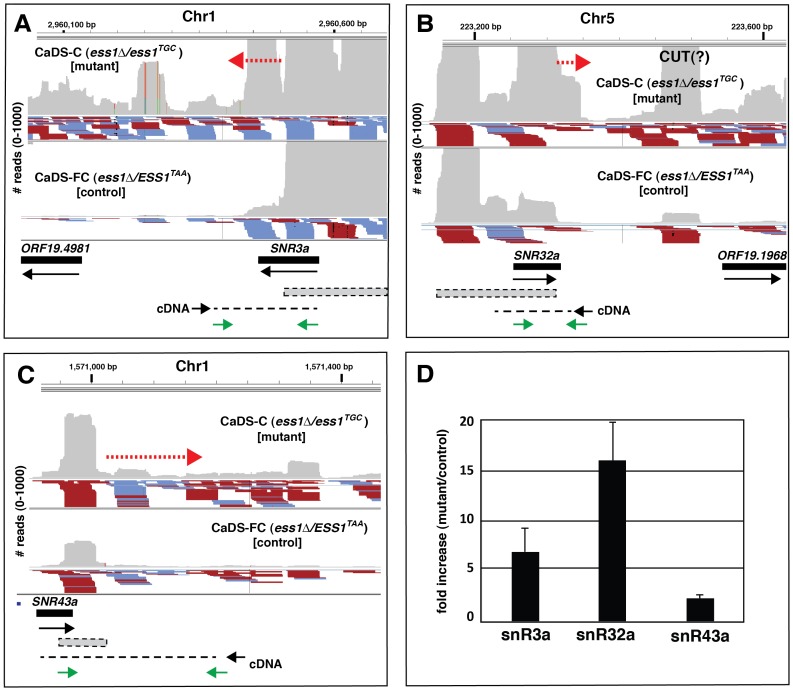
RNA-sequencing indicates transcription readthrough at SnoRNA loci. Results are visualized using Integrated Genome Viewer. The total number of RNA-sequencing reads (y-axis) and their position along the chromosome (x-axis) is indicated in grey. Forward oriented reads are indicated in red; reverse oriented reads are indicated in blue. Not all reads (blue, red) are visible. Results for the CaEss1 *ts*-mutant strain are in the upper panels, and results for the isogenic control are in the lower panels (**A**–**C**). Solid bars indicate the previously annotated gene positions, while the dotted (grey) boxes indicate the positions implied based on actual transcript data from RNA-sequencing of the the wild-type strain (*ESS1*/*ESS1*) (not shown) and the control strain (lower panels). A putative CUT is indicated in panel (B). Approximate positions of primers used for strand-specific cDNA synthesis are shown (black), as are the positions of the primer sets (green) used for qRT-PCR in (D). The positions of likely readthrough transcription are identified by red dashed arrows. (**D**) Results of qRT-PCR to detect readthrough transcripts for different snoRNA genes (x-axis), expressed as a fold-change (y-axis) of *ts*-mutant (CaDS-C) over isogenic control (CaDS-FC), normalized to *ACT1*. For RNA-sequencing, cell growth conditions are listed in Table 2 (shift to 42°C, then serum induction at 42°C), for qRT-PCR, samples were serum-induced at 37°C.

Each of the five H/ACA-class genes listed above contained an abundance of sequence reads (>30, but more often in the hundreds) immediately downstream of the putative termination site in the mutant background, suggesting possible transcription readthrough. The IGV profiles for three of these genes are shown (**Figure 7** **, A**–**C)**. RNA sequence reads in the forward direction (red) and reverse direction (blue) are indicated as well as the overall number of reads (grey). For the control (*ess1*Δ/*ESS1^TAA^*) (**Figure 7A**–**C**) and wild-type (*ESS1*/*ESS1*) (data not shown) strains, the large number of reads is likely to reveal the actual position of the snoRNA transcription units (dotted grey bars) more accurately than sequence annotations (solid bars) [68]–[70]. The IGV also reveals examples of increased transcript abundance in the mutant cells for some snoRNAs, e.g. *SNR332a*, *SNR43a* (**Figure 7B,C**), and a protein-coding gene (*ORF19.1968*) and a non-coding transcript (CUT) (**Figure 7B**).

To confirm the presence of snoRNA readthrough transcripts, we carried out qRT-PCR (**Figure 7D**). cDNA synthesis was done using strand-specific primers, so only transcripts in the forward (“sense) direction relative to the snoRNA transcription units would serve as templates. Primer sets for amplification consisted of one primer within the snoRNA gene, and the other downstream of the putative termination site (**Figure 7A**–**C**), therefore, only extended (readthrough) transcripts would be detected. The results show a 2–15 fold increase in the amount of readthrough transcription in the CaEss1 *ts*-mutant cells relative to isogenic control cells (**Figure 7D**). In summary, both the RNA-seq and qRT-PCR data indicate that CaEss1 is important for efficient termination of at least some snoRNA genes in *C. albicans*.

**Figure 8:**
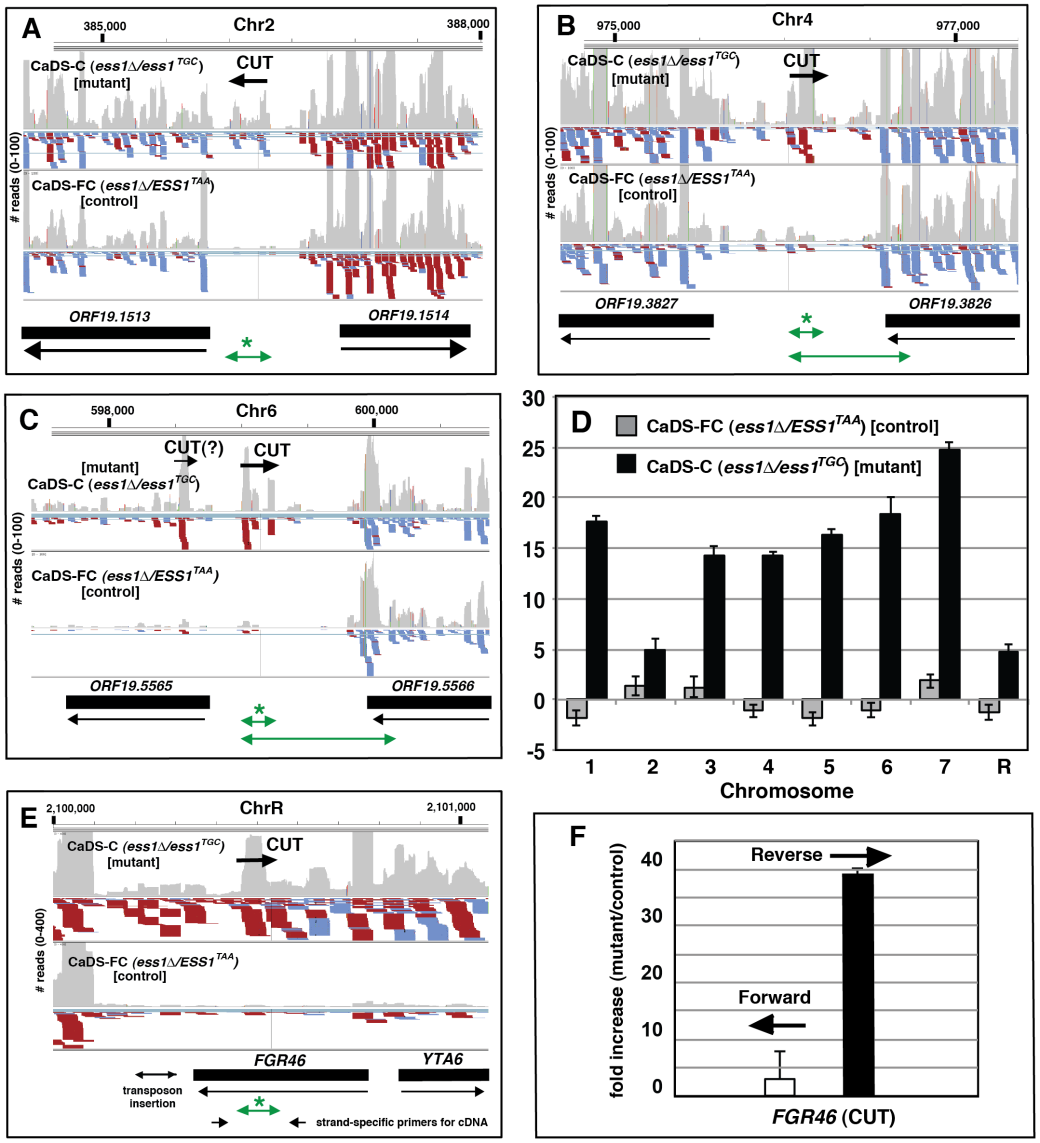
RNA-sequencing reveals widespread cryptic transcription in CaEss1 *ts*-mutant cells. Results are shown using Integrated Genome Viewer as described in the legend to Figure 7. The positions of cryptic unstable transcripts (CUTs) are identified on representative chromosomes (**A**–**C**). (**D**) Results of qRT-PCR to detect CUTs (examples for each chromosome) expressed as a fold-change over wild-type (CaDS-B5) (*ESS1*/*ESS1*), normalized to *ACT1.* The approximate positions of the qRT-PCR products in chromosome 2, 4 and 6 samples in (D) are indicated by the short green arrows marked by an asterisk (*). The longer products indicated by long green arrows (**B, C**) did not amplify, indicating that the CUT signal is not likely due to a readthrough product from nearby open reading frames (ORF). cDNA synthesis was primed using a mixture of random hexamers and oligo(dT). Cell growth conditions were as listed in Table 2 (shift to 42°C, then serum induction at 42°C). (**E**) Prominent CUT identified within open reading frame of *FGR46*, which has been implicated in filamentation. (**F**) CUT is in reverse orientation relative to the *FGR46* ORF as shown by strand-specific cDNA synthesis followed by qRT-PCR. Results are expressed as fold-change of *ts*-mutant (CaDS-C) over isogenic control (CaDS-FC), normalized to *ACT1*. Cells were serum-induced at 37°C. cDNA synthesis was primed using strand-specific primers that would reverse transcribe forward (UP044) or reverse (UP043) transcripts. For details, see Materials and Methods.

To determine if CaEss1 represses cryptic transcription we used IGV to compare the transcription profiles of the *ts*-mutant and its isogenic control, focusing on the intergenic regions along all of the chromosomes. In the mutant cells, intergenic transcription was rampant, with large amounts detected on all eight chromosomes. Examples are shown from chromomes 2, 4 and 8 (**Figure 8A**–**C**). Intergenic transcripts that lie between divergent genes are probably CUTs, since they cannot be due to readthrough transcription from neighboring genes (*e.g*. **Figure 8A**). Using both short and long qRT-PCR to characterize other potential CUTs revealed that only the short products (marked with an asterisk in **Figure 8B,C** `) were amplified (**Figure 8D**, and data not shown), indicating the RNA-seq reads are not due to readthrough transcription. These data suggest that in CaEss1 mutants, cryptic promoters are activated and/or cryptic RNAs are stabilized. CUTs from each of the eight chromosomes were easily detected by qRT-PCR (*e.g.* **Figure 8D**), indicating that CUT expression is widespread in CaEss1 mutant cells.

Many putative CUTs were also found within open reading frames, often in the antisense direction. One example is a potential antisense CUT within the open reading frame of *FGR46* (**Figure 8E**), a gene implicated in filamentation by a transposon insertion screen[71]. The reverse orientation of this CUT relative to the *FGR46* ORF was confirmed by strand-specific qRT-PCR (**Figure 8F**). Thus, it is possible that under conditions where Ess1 levels are strongly reduced, CUT induction might enhance or suppress expression of genes important for virulence. Additional analyses will be needed to fully explore the information in the RNA-sequencing datasets and determine the effect(s) of CaEss1 mutation on expression of virulence-related genes. Thus far, our findings are consistent with a conserved role for CaEss1 in transcription by RNA polymerase II.

## Conclusions

In this study, we showed that the *ESS1* gene is essential for growth in *C. albicans*. This result independently confirms earlier findings [52], but used more rigorous methods that included better controls and considered genomic sequence information that was not previously available. In contrast, the prior finding that reducing gene dosage by half (*ess1*Δ/*ESS1*) prevents morphogenetic switching, which is required for virulence *in vivo* [60], was not confirmed in the present study. The most likely explanation is that a background mutation(s) in the isolate of CAI4 used in the prior work caused the defect in filamentation. Thus, like other studies [72], [73], our results make clear the importance of reconstituted isogenic controls, appropriate marker gene systems, and the dangers of isolate-to-isolate variability between supposedly identical strains. The results of transcriptome analysis of CaEss1 mutants support the idea that regulation of RNA polymerase II by Ess1 is conserved between the evolutionarily distant species *S. cerevisiae* and *C. albicans*. Further studies will be needed to determine whether transcriptional defects are responsible for growth and morphogenetic switching phenotypes in CaEss1 mutants, and whether the mechanism by which Ess1 functions in transcription in *C. albicans* is similar to that in *S. cerevisiae* [43], [49]. With respect to *C. albicans* Ess1 as an antifungal drug target, data from this study suggest that elimination or strong inhibition of Ess1 enzyme activity will prevent growth. Given that Ess1 is essential not only in *C. albicans*, but also in *A. nidulans* [54], and is required for virulence in *C. neoformans* [53], it is possible that inhibitors of Ess1 could potentially be developed into broad-spectrum antifungal agents.

## Materials and Methods

### *C. albicans* strains, media, and transformation

All parent strains were grown in YPD media while the engineered derivative strains were grown in their respective selective media [74], unless otherwise specified. To select for *URA3* loop-out events, cells were plated on YPD supplemented with 1 mg/ml 5-FOA [75]. In addition to the descriptions below, please refer to the tables for details on primers (**Table 4**), plasmids (**Table 5**) and strains (**Table 6**) used in this study. Transformations were performed according to the lithium acetate protocol [76].

**Table 4:** Primers used in this study.

OW1075	tattaaatt**gcggccgc**TAGAGACAAGTTGTATAGATTTGG	Forward primer to amplify sequence downstream of *CaESS1* (+572 to +1265) with NotI ends
OW1081	aagctaata**gcggccgc**TGTATTTTTGTTCACGGAAT	Reverse primer to amplify sequence downstream of *CaESS1* (+572 to +1265) and (+729 to +1265) with NotI ends
OW221	CAATGACGGGAAACGTTCCG	Reverse primer for allele-specific PCR
OW769	**AAG** GTATTGAATGCATACATT**GAC**	Forward primer to detect helix mutations
OW770	GAAGTATTGAATGCATACATTGCG	Forward primer to detect wild-type CaEss1
OW1099	TTCTTTTTGCAGTAGTATATC**CAT**ATGGCATCGACATCAACAGGC	Mutagenesis primer to introduce NdeI site at ATG (*CaESS1* start codon)
OW1415	GCCGACGCCATTGTCGATTTAG	Forward primer to test expression of *HIS1*
D25	CCTGAGCCGCTAAAACACCTTGAA	Reverse primer to test expression of *HIS1*
OW1100	AGTGGTGTCCATATCCTCCAAAGAACAG**C**AT**GC**ATCAAGATATTGGAGTTTGATG	Mutagenesis primer to introduce SphI site at TAA (*CaESS1*stop codon)
OW1354	CGATGATCATG**CAT**ATGGCATCGACATCAACAGGCTTAC	Forward primer to amplify *CaESS1* sequence with NdeI site
OW1355	GTACGTA**GC**AT**G**CTGTTCTTTGGAGGATATGGACACCACT	Reverse primer to amplify *CaESS1* sequence with Sph1 site
OW216	CCAGATGGTATAAGTAGAAC	Forward primer to amplify *CaESS1* region with native stop codon (TAA)
OW1231	AAGCATGTAAAACATACGAT	Reverse primer to amplify *CaESS1* region with native stop codon (TAA)
OW749	**CTGGTGGTAAGAACGGTCAAGGTGAACCAGCTAG** AGTTAGAGTTTCTCATTTGTTGATC	Forward primer to amplify downstream portion of the linker swap
OW748	**CCTTGACCGTTCTTACCACCAGAAGAAGAGTTGT** CCCAAGACGACTCATTGG	Reverse primer to amplify upstream portion of the linker swap
OW751	**G** GTATTGAATGCATACATT**GAC**AAGTTT**GAA**AACAATGGTTACAAGCCACTTGTGAATGAG	Forward primer to amplify downstream portion of the helix mutant
OW750	**C** AAACTT**GTC**AATGTATGCATTCAATAC**CTT**TTTGTCAGTGCCATAAGGTGGGTC	Reverse primer to amplify upstream portion of the helix mutant
OW1224	CGCCAGGGTTTTCCCAGTCACGAC	Forward primer to amplify upstream portion of the helix mutant/linker swap
OW1233	GAGCGGATAACAATTTCACACAGG	Reverse primer to amplify downstream portion of the helix mutant/linker swap
D1	gtgctgaaagagaaattgtcagag	Forward primer to amplify *ACT1*
D2	aaacctaaatcagctggtctgaac	Reverse primer to amplify *ACT1*
D3	agccaagagggttattgatgttag	Forward primer to amplify *URA3*
D4	tctaatccaactccaggtgtcata	Reverse primer to amplify *URA3*
D6	cttcacaaagaagtggattttcctc	Forward primer to amplify *APE2*
D7	ggaaagaaagcggaaagaaaga	Reverse primer to amplify *APE2*
D31	tattaaatt**gcggccgc**GATCCATCTGGTCCCATGGCTTC	Forward primer to amplify the sequence downstream of *CaESS1* (+729 to +1265) with NotI ends
D32	GGAAAGAATATTGAGTGGTGAGGT	Forward primer to amplify *CaESS1*
D33	TACTGACTTCTCCAACATGCAAA	Reverse primer to amplify *CaESS1*
UP043	TGGAATCTGTTTTGTTAG	FGR46 GSP (detect reverse CUT)
UP044	TAGCAAGCAAGAGATTTTGAAG	FGR46 GSP (detect coding region)
D52	GAACAAGGGCAGAAAGAAGAAGT	FGR46 rt-PCR (F)
D53	GCAATCAGACGCAGTTGAAATA	FGR46 rt-PCR (R)
UP045	CTTAAATAAATTTCTTTATTATCGTTC	snR3a GSP primer
UP046	ATTGATCTTGGGTGCAGC	snR3a rt-PCR (F)
UP047	TCATCTACAAAAGTCCGCTGT	snR3a rt-PCR (R)
UP051	CTAATTGGTAAAGAG	snR32a GSP primer
UP052	GCGCCTGGTGTTCACAT	snR32a rt-PCR (F)
UP053	AGAGAATTGGTAAAACTGTAGTCACAAC	snR32a rt-PCR (R)
UP055	CTGTTATGGCATTGGAGC	snR43a GSP primer
UP056	GGTTCTGGTGAAGGAGCAAA	snR43a rt-PCR (F)
D111	TGGAGCTTGGATAAATAGTGTGAC	snR43a rt-PCR (R)
UP058	TTGAAAGTGGTTTGGTC	ACT1 GSP primer

Lower case sequences added to obtain appropriate length. Bold indicates a mutation or restriction site.

**Table 5:** Plasmids used in this study.

Plasmid Name	SelectableMarker	Plasmid Content of Interest
pDS426(pm)	*URA3*	*pCaESS1*(−255 to 0)−*Caess1^hm^*−*terCaESS1*(+535 to +735)
pDS426(sw)	*URA3*	*pCaESS1*(−255 to 0)−*Caess1^sw^*−*terCaESS1*(+535 to +735)
pDS413(pm)	*HIS3*	*pCaESS1*(−255 to 0)−*Caess1^hm^*−*terCaESS1*(+535 to +735)
pDS413(sw)	*HIS3*	*pCaESS1*(−255 to 0)−*Caess1^sw^*−*terCaESS1*(+535 to +735)
pDS(b)Leu	*LEU2*	*pCaESS1*(−255 to 0)−*Caess1^hm^*−*terCaESS1*(+535 to +735)−*LEU2*
pDS(b)His	*HIS1*	*pCaESS1*(−255 to 0)−*Caess1^hm^*−*terCaESS1*(+535 to +735)−*HIS1*
pDS(c)Leu	*LEU2*	*pCaESS1*(−255 to 0)−*Caess1^hm^*−*terCaESS1*(+535 to +735)−*LEU2*−d.s.*CaESS1*(+572 to +1265)
pDS(c)His	*HIS1*	*pCaESS1*(−255 to 0)−*Caess1^hm^*−*terCaESS1*(+535 to +735)−*HIS1*−d.s.*CaESS1*(+572 to +1265)
pDS(d)Leu	*LEU2*	*pCaESS1*(−255 to 0)−*Caess1^hm^*−*terCaESS1*(+535 to +735)−*LEU2* −d.s.*CaESS1*(+572 to +1265) *no NdeI site in vector backbone
pDS(d)His	*HIS1*	*pCaESS1*(−255 to 0)−*Caess1^hm^*−*terCaESS1*(+535 to +735)−*HIS1*−d.s.*CaESS1*(+572 to +1265) *no NdeI site in vector backbone
pDS(e)Leu	*LEU2*	*pCaESS1*(−255 to 0)−*Caess1^hm^*−*terCaESS1*(+535 to +735)−*LEU2* −d.s.*CaESS1*(+572 to +1265) *NdeI site introduced at ATG start codon
pDS(e)His	*HIS1*	*pCaESS1*(−255 to 0)−*Caess1^hm^*−*terCaESS1* (+535 to +735)−*HIS1*−d.s.*CaESS1*(+572 to +1265) *NdeI site introduced at ATG start codon
pDS(f)Leu	*LEU2*	*pCaESS1*(−255 to 0)−*Caess1^hm/TGC^*−*terCaESS1*(+535 to +735) −*LEU2* −d.s.*CaESS1*(+572 to+1265) *NdeI site introduced at ATG start codon *SphI site (TGC) introduced at TAA stop codon
pDS(g)Leu	*LEU2*	*pCaESS1*(−255 to 0)−*Caess1^TGC^*−*terCaESS1*(+535 to +735)−*LEU2* −d.s.*CaESS1*(+572 to +1265) *NdeI site introduced at ATG start codon *SphI site (TGC) introduced at TAA stop codon
pDS(x)His	*HIS1*	*pCaESS1*(−255 to 0)−*HIS1*−d.s.*CaESS1*(+572 to +1265) *no NdeI site at ATG start codon *591bp left as *pHIS1* while 393bp sequence upstream was removed during plasmid construction
pDS(h)Leu	*LEU2*	*pCaESS1*(−255 to 0)−*Caess1^hm^*−*terCaESS1*(+535 to +735)−*LEU2* −d.s.*CaESS1*(+729 to +1265)
pDS(h)His	*HIS1*	*pCaESS1*(−255 to 0)−*Caess1^hm^*−*terCaESS1* (+535 to +735)−*HIS1*−d.s.*CaESS1*(+729 to +1265)

**Table 6:** Strains used in this study.

Strain name	Parent strain	Genotype	Source
SC5314	clinical isolate	Wild-type	Fonzi and Irwin (1993)
CAI4	SC5314	*ura3*Δ:: *imm434/ura3*Δ*:: imm434*	Fonzi and Irwin (1993)
CaGD1	CAI4 (*)	*ESS1*/*ess1*Δ*:: hisG-URA3-hisG ura3:: imm434*/*ura3:: imm434*	Devasahayam et al., 2002
CaGD2	CaGD1	*ESS1*/*ess1*Δ*:: hisG ura3:: imm434*/*ura3:: imm434*	Devasahayam et al., 2002
R6	CaGD2	*ESS1*/*ESS1:: hisG-URA3-hisG ura3:: imm434*/*ura3:: imm434*	Devasahayam & Hanes, unpublished
R6*	R6	*ESS1*/*ESS1:: hisG ura3:: imm434*/*ura3:: imm434*	this study
CaGD2*+pLUBP (−/+) *URA3* at native	CaGD2	*ESS1*/*ess1*Δ*:: hisG URA3*/*ura3:: imm434*	this study
R6*+pLUBP (+/+) *URA3* at native	R6*	*ESS1*/*ESS1:: hisG URA3*/*ura3:: imm434*	this study
CaGD2*+CIp10 (−/+) *URA3* at *RPS1*	CaGD2	*ESS1*/*ess1*Δ*:: hisG ura3:: imm434*/*ura3:: imm434 rps1*Δ*:: URA3/RPS1*	this study
R6*+CIp10 (+/+) *URA3* at *RPS1*	R6*	*ESS1*/*ESS1:: hisG ura3:: imm434*/*ura3:: imm434 rps1*Δ*:: URA3/RPS1*	this study
CAI4*+pLUBP *URA3* at native	CAI4 (*)	*URA3/ura3*Δ*:: imm434*	this study
CAI4*+CIp10 *URA3* at *RPS1*	CAI4 (*)	*ura3*Δ:: *imm434/ura3*Δ*:: imm434 rps1*Δ*:: URA3/RPS1*	this study
CAI12	CAI4	*URA3/ura3*Δ*:: imm434*	Porta et al., 1999
CaDS-B0.5	SN87	*ESS1*/*ESS1:: C.m.LEU2 leu2*Δ/*leu2*Δ *his1*Δ/*his1*Δ *URA3/ura3*Δ*:: imm434*	this study
CaDS-B5	CaDS-B0.5	*ESS1:: C.d.HIS1*/*ESS1:: C.m.LEU2 leu2*Δ/*leu2*Δ *his1*Δ/*his1*Δ *URA3/ura3*Δ*:: imm434*	this study
CaDS-B5.5	CaDS-B0.5	*ess1*Δ*:: C.d.HIS1*/*ESS1:: C.m.LEU2 leu2*Δ/*leu2*Δ *his1*Δ/*his1*Δ *URA3/ura3*Δ*:: imm434*	this study
CaDS-87-2.3	SN87	*ess1*Δ*:: C.d.HIS1*/*ESS1 leu2*Δ/*leu2*Δ *his1*Δ/*his1*Δ *URA3/ura3*Δ*:: imm434*	this study
CaDS-C	CaDS-87-2.3	*ess1*Δ*:: C.d.HIS1*/*ess1^TGC^:: C.m.LEU2 leu2*Δ/*leu2*Δ *his1*Δ/*his1*Δ *URA3/ura3*Δ*:: imm434*	this study
CaDS-FC	CaDS-C	*ess1*Δ*:: C.d.HIS1*/*ess1^TAA^:: C.m.LEU2 leu2*Δ/*leu2*Δ *his1*Δ/*his1*Δ *URA3/ura3*Δ*:: imm434*	this study
CaDS-E	SN87	*ESS1*/*ess1^hm^:: C.m.LEU2 leu2*Δ/*leu2*Δ *his1*Δ/*his1*Δ *URA3/ura3*Δ*:: imm434*	this study
CaDS-E5	CaDS-E	*ess1^hm^:: C.d.HIS1*/*ess1^hm^:: C.m.LEU2 leu2*Δ/*leu2*Δ *his1*Δ/*his1*Δ *URA3/ura3*Δ*:: imm434*	this study
CaDS-E5.10	CaDS-E	*ess1*Δ*:: C.d.HIS1*/*ess1^hm^:: C.m.LEU2 leu2*Δ/*leu2*Δ *his1*Δ/*his1*Δ *URA3/ura3*Δ*:: imm434*	this study
CaDS-51B-2	SN87	*ESS1-5′ape2:: C.d.HIS1*/*ESS1-5′ape2:: C.m.LEU2 leu2*Δ/*leu2*Δ *his1*Δ/*his1*Δ *URA3/ura3*Δ*:: imm434*	this study

(*C.d.*) *Candida dubliniensis;* (*C.m.*) *Candida maltosa*; (*5′*) promoter region truncated; (*hm*) helix mutant.

Abbreviations: (*p*) promoter region; (*ter*) termination region; (*sw*) linker swap; (*hm*) helix mutant; (d.s.) downstream sequence.

(*) may have acquired a mutation that affects filamentation as per this study.

### *C. abicans* strain construction

#### Insertion of the *URA3* gene at different genomic positions

For re-evaluating the *C. albicans* heterozygous *Caess1* mutant (CaGD1)[52] along with its control strain, R6 (*CaESS1/CaESS1*), the *URA3* gene was removed from the *CaESS1* locus using 5-FOA mediated loop-out events to create uracil auxotrophs CaGD2 (*Caess1*δ*/CaESS1*) and R6* (*CaESS1/CaESS1*) respectively. One copy of *URA3* was integrated at the native *URA3* locus by digesting pLUBP plasmid [77] with BglII and PstI restriction enzymes and transforming into the above strains (CaGD2 and R6*). Similarly, one copy of *URA3* was integrated at the *RPS1* locus in these strains by digesting CIp10 plasmid [78] with StuI restriction enzyme and transforming. *URA3* was also integrated into the parent strain CAI4* using the same pLUBP and CIp10 plasmids. (*) indicates a strain that may have acquired one or more mutations affecting filamentation as per this study.

#### Isogenic wild-type control strain, CaDS-B5 (*ESS1/ESS1*)

Plasmid pDS426(pm) is a derivative of pRS426 that carries the *CaESS1* promoter region (−255 to 0), *CaESS1* ORF (with helix point mutations) and *CaESS1* termination region (+535 to +735) between EcoR1 and BamH1 sites. The *LEU2* marker (from plasmid pSN40) or the *HIS1* marker (from plasmid pSN52) [62] was cloned into plasmid pDS426(pm) between the BamH1 and Not1 sites. These plasmids were named pDS(b)Leu and pDS(b)His, respectively. Because in the *C. albicans* genome, *APE2* is just 210 bp downstream of *ESS1*, a 163 bp sequence (+572 to +735) was repeated on either side of the selectable markers to allow enough sequence for *CaESS1* termination and for the 5′ *APE2* promoter region. This was done by amplifying the downstream sequence of *CaESS1* (+572 to +1265) with NotI ends (primers OW1075 and OW1081), digesting and inserting into pDS(b)Leu and pDS(b)His, thus positioning the NotI-NotI downstream sequence after the markers. The resulting plasmids are pDS(c)Leu and pDS(c)His.

The pDS(c)Leu and pDS(c)His plasmids were digested with SacI and serially transformed into SN87 [62]. In the first step, one *CaESS1* allele was marked with the *LEU2* gene to generate strain CaDS-B0.5 (*ESS1: LEU2/ESS1*). In the next step, the second allele of *CaESS1* was marked with *HIS1* gene to generate a Leu+ His+ control strain CaDS-B5 (*ESS1: LEU2/ESS1: HIS1*). Allele-specific PCR (primer OW221 with OW769 or OW770) and DNA sequencing was used to identify clones in which the integrating DNA recombined at positions that generated the wild-type *CaESS1* ORF, (*i.e.* that excluded the helix mutations). The gene modifications were verified using junction-PCR and Southern blot hybridization (data not shown).

#### Heterozygous mutant strain, CaDS-B5.5 (*ess1*δ*/ESS1*)

The NdeI site in the backbone of the plasmid, pDS(c)His was removed by digestion with NdeI, Klenow treatment and re-ligation, to form pDS(d)His. The start codon (ATG) of *CaESS1* ORF in pDS(d)His was then converted to an NdeI site with the Change-IT Multiple Mutation Site Directed Mutagenesis Kit (USB) using the mutagenesis primer OW1099. The resulting pDS(e)His plasmid was digested with Nde1 and Spe1 (site 591 bp upstream of the *HIS1* marker), Klenow treated and re-ligated to generate a plasmid that lacked the *ESS1* ORF sequence. The resulting plasmid, pDS(x)His was digested with SacI and transformed into strain CaDS-B0.5 (*ESS1: LEU2/ESS1*) to generate a heterozygous mutant, CaDS-B5.5 (*ESS1: LEU2/ess1*δ*: HIS1*). The gene modifications were verified using junction-PCR and Southern blot hybridization (data not shown). In plasmid, pDS(x)His the *HIS1* marker (from the pSN52 plasmid) was left with a 591 bp promoter region. An additional 393 bp sequence that was upstream of the promoter region of *HIS1* is removed in this construct. This had no affect on *HIS1* expression by qRT-PCR (primers OW1415 and D25) in comparison to strain CaDS-B5 (*ESS1/ESS1*) (data not shown).

#### Temperature-sensitive strain, CaDS-C (*ess1*δ*/ess1^(TGC^*) and isogenic control

The plasmid pDS(x)His was digested with SacI and transformed into SN87 to generate CaDS-87-2.3 (*ess1*δ*/ESS1)*, which is auxotrophic for *LEU2*. The Nde1 site of the vector portion in plasmid pDS(c)Leu was removed (as done for pDS(c)His), to form pDS(d)Leu. Next, the start codon (ATG) and the stop codon (TAA) of *CaESS1* in plasmid pDS(d)Leu were changed to an NdeI site and a SphI site (primers OW1099 and OW1100, respectively) in two steps, to first form plasmid pDS(e)Leu and then plasmid pDS(f)Leu, respectively. The wild-type *CaESS1* sequence was amplified (primers OW1354 and OW1355) with flanking Nde1 and Sph1 sites, digested and inserted into the same sites of pDS(f)Leu to give pDS(g)Leu. The Sph1 site changes the stop codon from TAA to TGC. The pDS(g)Leu plasmid was digested with SacI and transformed into CaDS87-2.3 (*ess1*δ*/ESS1)* to make a prototrophic strain, CaDS-C (*ess1*δ*/ess1^TGC^*). This strain was verified using junction-PCR and Southern blot hybridization (data not shown). The control strain was generated by a novel strategy. About a 900 bp PCR fragment amplified (using primers OW216 and OW1231) from a wild-type background containing the normal termination codon (TAA) was introduced into CaDS-C (*ess1*δ*/Caess1^TGC^*). Transformants capable of growth at 42°C were selected, analyzed by PCR amplification and DNA sequencing of the *CaESS1* alleles and the mutation of TGC to TAA was confirmed. The resulting prototrophic strain CaDS-FC (*ess1*δ*/ESS1^TAA^*) produced Ess1 protein of the normal size (19 kDa) and was non-*ts.*

#### Linker-helix mutations in CaEss1

PCR overlap extension (with primer sets OW748-OW1224, OW749-OW1233, and OW1224-OW1233) was used to generate a fragment that encodes CaEss1 protein bearing the linker region of human Pin1 (codon optimized for *C. albicans* [79]. This linker-swap fragment was digested with EcoRI and BamHI sites and cloned into the same sites of pRS426 vector to form pDS426(sw). A similar strategy was used to generate a CaEss1 mutant bearing three amino acid substitutions (E44K, A51D, and K54E) in the linker helix (using primers OW750 and OW751). The helix mutant fragment was cloned into pRS426 vector to form pDS426(pm).

#### Helix mutants, CaDS-E (*ESS1/hm*), CaDS-E5 (*hm/hm*), and CaDS-E5.10 (*ess1*δ*/hm*)

To construct CaDS-E (*ESS1/hm*) strains, the plasmid, pDS(c)Leu was digested with SacI and transformed into SN87. Allele-specific PCR and DNA sequencing was used to verify the clones that recombined to include the helix mutations (*Caess1^hm^*). This strain is auxotrophic for *HIS1.* To construct prototrophic CaDS-E5 (*hm/hm*) strains, pDS(c)His was digested with SacI and transformed into CaDS-E (*ESS1/hm*). To construct CaDS-E5.10 (*ess1*δ*/hm*) strains, pDS(x)His was digested with SacI and transformed into CaDS-E(*ESS1/hm*). Mutants were verified using junction-PCR and Southern blot hybridization (data not shown).

#### *APE2* promoter mutant, CaDS-51B-2 (*5′ape2/*5′*ape2*)

A Not1-Not1 PCR fragment (primers D31 and OW1081) was cloned into plasmids pDS(b)Leu and pDS(b)His, to form plasmids pDS(h)Leu and pDS(h)His, respectively. This fragment (+729 to +1265) contained only 15 bp of 5′ genomic sequence upstream of *APE2* (and an upstream Not1 site). The plasmid was digested with SacI and transformed into SN87, in two steps to generate strain CaDS-51B-2 (*5*′*ape2/*5′*ape2*).

### Growth assays

Overnight cultures of either *S. cerevisiae* or *C. albicans* were diluted to an OD_600_ of 0.1 and grown with shaking at 30°C until midlog phase. Cultures were brought to an OD_600_ of 0.5 either by dilution or concentration by centrifugation. For *S. cerevisiae*, a 1∶5 dilution series of the strains were spotted onto plates containing selective media and incubated for two nights. For *C. albicans* strains, a 1∶3 dilution series was used, with cells spotted on YPD plates and incubated overnight.

### Filamentation assays

Overnight cultures of *C. albicans* were diluted to an OD_600_ of 0.25 (∼3×10^6^ cells/ml). From dilution, 2 µl of culture was spotted onto Spider media [80] and serum-containing media (4% FBS and 2% agar). The plates were incubated at 37°C for 4 days prior to being photographed. To test germ tube formation, a suspension of 10^5^–10^6^ cells/ml was made in liquid Spider medium [80], and incubated at 37°C for 2 hrs prior to microscopic observation.

### Western blot analysis

Overnight cultures were diluted to an OD_600_ of 0.1, grown to an OD_600_ of 0.5 and harvested by centrifugation. Protein extracts were prepared using yeast protein lysis buffer (50 mM HEPES, 140 mM NaCl, 1 mM EDTA, 1% Triton X and, 0.1% sodium deoxycholate) with proteinase inhibitor cocktail (ethanolic protease inhibitors with 0.25 M PMSF and 0.7 mg/ml pepstatin/aqueous protease inhibitors with 0.1 mg/ml leupeptin, 1 mg/ml soybean trypsin inhibitor, 0.1 mg/ml aprotinin in 10 mM Tris, pH7.5, 20 mM benzamidine, 10 mM sodium vanadate and, 500 mM sodium fluoride). A 15% polyacrylamide gel was run at 100 V for about 2.5 hrs and transferred to a PVDF Transfer Membrane (Millipore) overnight at 32 V at 4°C. A 1∶500 dilution of rabbit α-CaEss1 polyclonal antibodies (Applied Biosystems) was used as primary antibody and a 1∶25,000 dilution of horseradish peroxidase-linked donkey anti-rabbit IgG antibody (GE Healthcare) was used as the secondary antibody. Purified CaEss1 made in *E. coli* [50] was used to generate CaEss1 polyclonal antibodies at Applied Biosystems. Detection was done using the ECL-Plus Western Blotting Detection System (GE Healthcare) and exposure to X-ray film (Kodak).

### High-throughput RNA sequencing

#### RNA and protein preparation

For the *ts*-readthrough mutant and control strain CaDS-C (*Caess1*δ*/Caess1^TGC^*) and CaDS-FC (*Caess1*δ*/CaESS1^TAA^*) the following protocol was used. Cultures (10 ml) were grown overnight in selective media at 30°C, diluted 10-fold in YPD, and grown overnight again to an OD_600_ of 9–11 [81]. Cultures were diluted 30 times in pre-warmed (42°C), pre-shaken fresh YPD (1 ml in 29 ml YPD)[81], and cells were grown at 42°C with shaking. After 1 hr, 2 ml of culture was collected for protein preparation as above and the remaining culture diluted 2-fold in pre-warmed (42°C), pre-shaken YPD+20% FBS (20 ml culture in 20 ml YPD+20% FBS) for serum induction. After 2 hr incubation at 42°C, cultures were collected by centrifugation for protein preparation (2 ml) and RNA extraction (10 ml). At extraction the cells were at an OD_600_ of 0.4. RNA was extracted by the hot phenol method [82]. For all other strains the following protocol used. Overnight cultures in YPD were grown to an OD_600_ of 9–11, and diluted 20-fold in pre-warmed (37°C), pre-shaken fresh YPD+10% FBS (1 ml culture in 19 ml YPD+10% FBS) [81]. After a 2 hr incubation at 37°C, 10 ml of culture (OD_600_ of 0.8) was collected for RNA extraction.

RNA was quantified using a Nanodrop Spectrophotometer and aliquots of 10 µg of RNA subjected to DNase digestion (Epicentre) for 45 min at 37°C. Digested RNA was purified using an RNeasy Mini Kit (Qiagen). 400 ng of RNA was fractionated by agarose (1%) gel electrophoresis to confirm integrity (data not shown). For the *ts*-readthrough strain and its isogenic control, protein extracts before and after the temperature shift and serum induction were analyzed by Western blot analysis to confirm reduced amount of Ess1 protein at the restrictive temperature (42°C).

#### Library preparation and high-throughput sequencing

RNA samples (1 µg in 10 µl of RNase free water) were sent for library preparation and sequencing to off-site facilities (Weill Cornell Medical College, NY and Centrillion Biosciences, CA). At Cornell, from 1 µg of purified RNA, ribosomal RNA was removed using Ribo-Zero rRNA Removal Kit (Epicenter). Illumina-compatible, barcoded, strand-specific, cDNA libraries were prepared using ScriptSeq mRNA-Seq Library Preparation Kit and RNA-Seq Barcode Primers (Epicenter). Illumina high-throughput sequencing was performed on the libraries using the HiSeq2000 sequencer running at 58 cycles (per lane) resulting in one-end read lengths of about 51 bp. At Centrillion, a similar approach was taken to prepare Illumina-compatible, barcoded, strand-specific, cDNA libraries with the exceptions of using the RiboMinus Eukaryote Kit (Invitrogen) for removal of ribosomal RNA and the ScriptSeq V2 RNA-Seq Library Preparation Kit (Epicenter). The ScriptSeq V2 Kit is an improved version of the ScriptSeq Kit and uses the same basic principal for library construction. Here the HiSeq2000 sequencer was run at 58×2 cycles (per lane) resulting paired-end read lengths of about 75 bp.

#### RNA sequence analysis

The raw FastQ data files were aligned to the *C. albicans* genome (Assembly 21) [83] obtained from Candida Genome Database (http://www.candidagenome.org/) using Bowtie/TopHat short sequence read alignment software programs [84], [85]. Each strain resulted in a large number of reads mapped to the genome (**Table 2**). The output files (in SAM format) were converted to BAM files using SAM Tools [86]. The BAM files were visually analyzed on Integrative Genomics Viewer (http://www.broadinstitute.org/igv/) and/or GenomeView (http://genomeview.org/). The files were quantitatively analyzed using the Galaxy server (https://main.g2.bx.psu.edu/), an open access platform for high-throughput data analysis. For example, the SAM or BAM files were used as Cuffdiff (a part of the open-source Cufflinks software package) [85] input files to identify differential expression of transcripts genome-wide among test strains and controls.

### Quantitative reverse transcription real-time PCR

Overnight cultures were diluted to an OD_600_ of 0.5 and grown to an OD_600_ of 0.8–1.2 and harvested. RNA was extracted using the hot phenol method [82] and subjected to DNase digestion (Epicenter) for 45 min at 37°C. From 1 µg of RNA, cDNA was synthesized using the First Strand cDNA Synthesis Kit for Real-Time PCR(USB) using mixture of oligo dT and random hexamers. In some sets of experiments cDNA was synthesized using individual cDNA synthesis reagents purchased from USB/Affimetrix. Quantitative real-time PCR was performed in the ABI2000 RT-PCR machine using the HotStart-IT SYBR Green qPCR Master Mix (USB). In some sets of experiments real-time PCR was carried out using the Fermentus SYBR Green Master Mix (Life Sciences). All results were normalized agains the same internal control gene, *ACT1*. qRT-PCR calculations were done as per Yu *et al*., [87] where the quantitative real-time PCR cycle numbers relative to *ACT1* were summed for each biological replicate and normalized to the summed average of all samples within a given experiment. The re-normalized values were then used for average and standard deviation calculations. This method corrects for trial-to-trial variability. To calculate the fold difference between samples, the *ACT1*-normalized (ΔCt) values for the FC (WT/-) and C (ts/−) data sets were normalized against the WT/WT (B5) data (to obtain ΔΔCt), and the following calculation was performed: fold difference = 2^−ΔΔCt^. The values obtained were averaged, and a standard deviation calculated.
